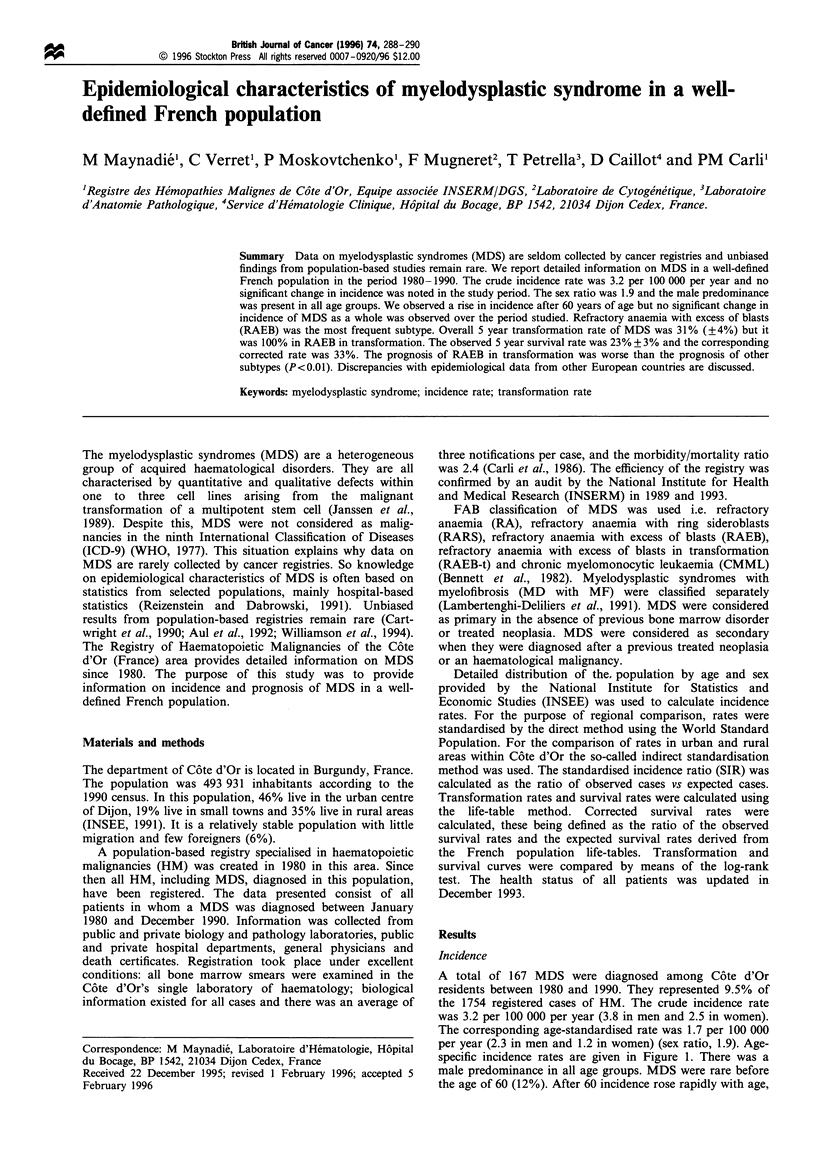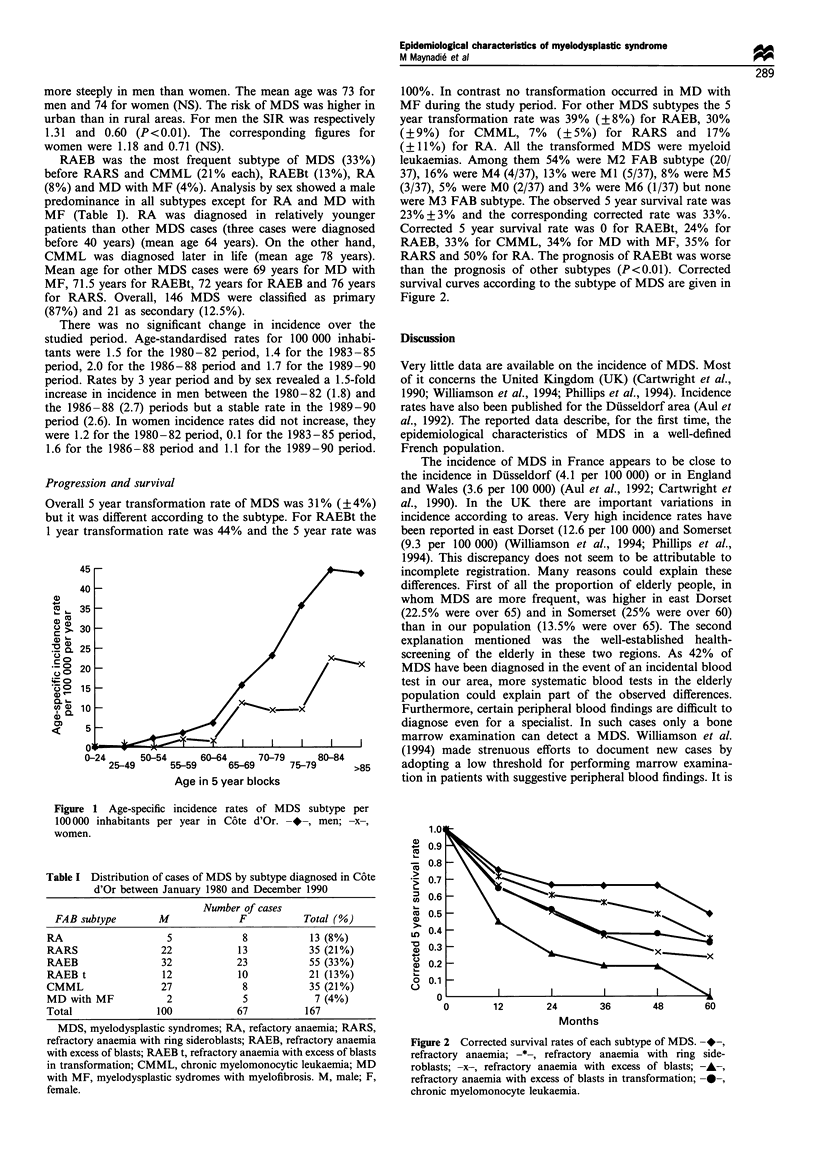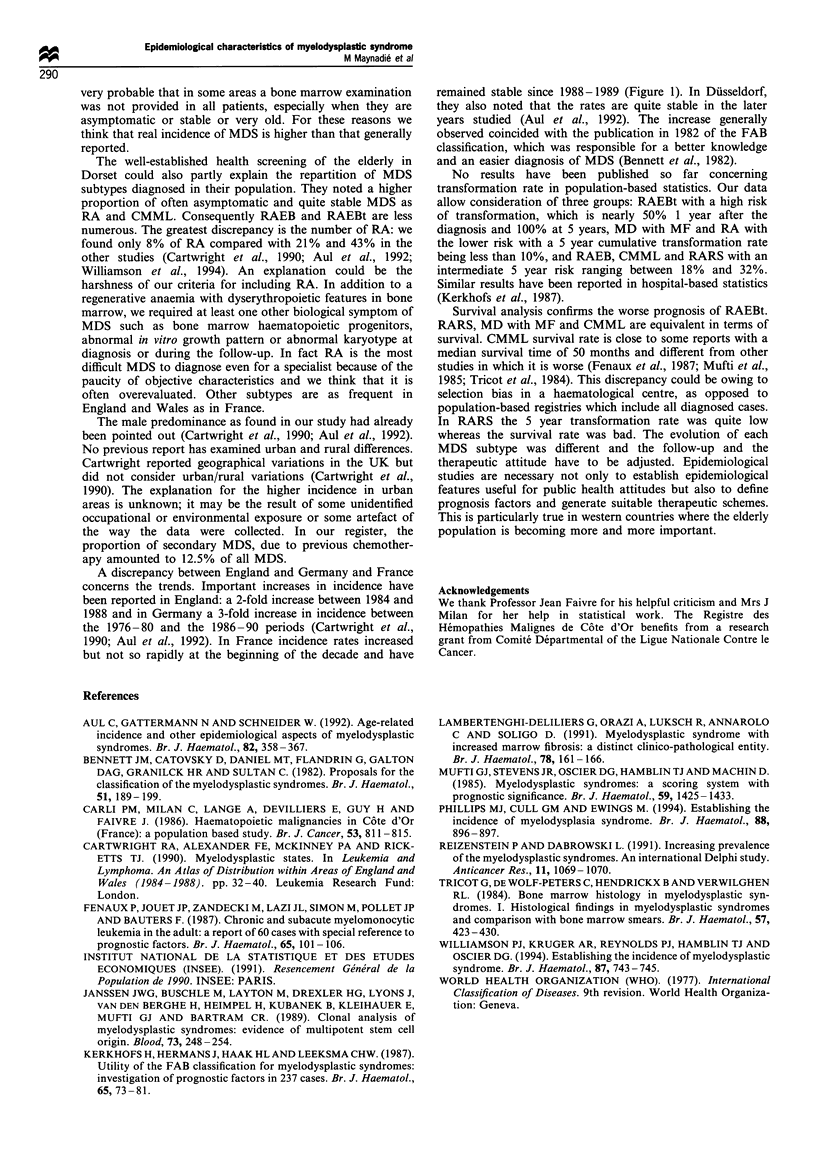# Epidemiological characteristics of myelodysplastic syndrome in a well-defined French population.

**DOI:** 10.1038/bjc.1996.354

**Published:** 1996-07

**Authors:** M. Maynadié, C. Verret, P. Moskovtchenko, F. Mugneret, T. Petrella, D. Caillot, P. M. Carli

**Affiliations:** Registre des Hémopathies Malignes de Côte d'Or, Equipe associée INSERM/DGS, Dijon, France.

## Abstract

Data on myelodysplastic syndromes (MDS) are seldom collected by cancer registries and unbiased findings from population-based studies remain rare. We report detailed information on MDS in a well-defined French population in the period 1980-1990. The crude incidence rate was 3.2 per 100000 per year and no significant change in incidence was noted in the study period. The sex ratio was 1.9 and the male predominance was present in all age groups. We observed a rise in incidence after 60 years of age but no significant change in incidence of MDS as a whole was observed over the period studied. Refractory anaemia with excess of blasts (RAEB) was the most frequent subtype. Overall 5 year transformation rate of MDS was 31% (+/- 4%) but it was 100% in RAEB in transformation. The observed 5 year survival rate was 23% +/- 3% and the corresponding corrected rate was 33%. The prognosis of RAEB in transformation was worse than the prognosis of other subtypes (P < 0.01). Discrepancies with epidemiological data from other European countries are discussed.


British Journal of Cancer (1996) 74, 288-290
© 1996 Stockton Press All rights reserved 0007-0920/96 $12.00

### Epidemiological characteristics of myelodysplastic syndrome in a well-defined French population

M Maynadié[1], C Verret[1], P Moskovtchenko[1], F Mugneret[2], T Petrella[3], D Caillot[4] and PM Carli[1]

*[1]Registre des Hémopathies Malignes de Côte d'Or, Equipe associée INSERM/DGS, [2]Laboratoire de Cytogénétique, [3]Laboratoire d'Anatomie Pathologique, [4]Service d'Hématologie Clinique, Hôpital du Bocage, BP 1542, 21034 Dijon Cedex, France.*

**Summary** Data on myelodysplastic syndromes (MDS) are seldom collected by cancer registries and unbiased findings from population-based studies remain rare. We report detailed information on MDS in a well-defined French population in the period 1980–1990. The crude incidence rate was 3.2 per 100 000 per year and no significant change in incidence was noted in the study period. The sex ratio was 1.9 and the male predominance was present in all age groups. We observed a rise in incidence after 60 years of age but no significant change in incidence of MDS as a whole was observed over the period studied. Refractory anaemia with excess of blasts (RAEB) was the most frequent subtype. Overall 5 year transformation rate of MDS was 31% ($\pm$4%) but it was 100% in RAEB in transformation. The observed 5 year survival rate was 23% $\pm$ 3% and the corresponding corrected rate was 33%. The prognosis of RAEB in transformation was worse than the prognosis of other subtypes ($P<0.01$). Discrepancies with epidemiological data from other European countries are discussed.

**Keywords:** myelodysplastic syndrome; incidence rate; transformation rate

The myelodysplastic syndromes (MDS) are a heterogeneous group of acquired haematological disorders. They are all characterised by quantitative and qualitative defects within one to three cell lines arising from the malignant transformation of a multipotent stem cell (Janssen *et al.*, 1989). Despite this, MDS were not considered as malignancies in the ninth International Classification of Diseases (ICD-9) (WHO, 1977). This situation explains why data on MDS are rarely collected by cancer registries. So knowledge on epidemiological characteristics of MDS is often based on statistics from selected populations, mainly hospital-based statistics (Reizenstein and Dabrowski, 1991). Unbiased results from population-based registries remain rare (Cartwright *et al.*, 1990; Aul *et al.*, 1992; Williamson *et al.*, 1994). The Registry of Haematopoietic Malignancies of the Côte d'Or (France) area provides detailed information on MDS since 1980. The purpose of this study was to provide information on incidence and prognosis of MDS in a well-defined French population.

### Materials and methods

The department of Côte d'Or is located in Burgundy, France. The population was 493 931 inhabitants according to the 1990 census. In this population, 46% live in the urban centre of Dijon, 19% live in small towns and 35% live in rural areas (INSEE, 1991). It is a relatively stable population with little migration and few foreigners (6%).

A population-based registry specialised in haematopoietic malignancies (HM) was created in 1980 in this area. Since then all HM, including MDS, diagnosed in this population, have been registered. The data presented consist of all patients in whom a MDS was diagnosed between January 1980 and December 1990. Information was collected from public and private biology and pathology laboratories, public and private hospital departments, general physicians and death certificates. Registration took place under excellent conditions: all bone marrow smears were examined in the Côte d'Or's single laboratory of haematology; biological information existed for all cases and there was an average of three notifications per case, and the morbidity/mortality ratio was 2.4 (Carli *et al.*, 1986). The efficiency of the registry was confirmed by an audit by the National Institute for Health and Medical Research (INSERM) in 1989 and 1993.

FAB classification of MDS was used i.e. refractory anaemia (RA), refractory anaemia with ring sideroblasts (RARS), refractory anaemia with excess of blasts (RAEB), refractory anaemia with excess of blasts in transformation (RAEB-t) and chronic myelomonocytic leukaemia (CMML) (Bennett *et al.*, 1982). Myelodysplastic syndromes with myelofibrosis (MD with MF) were classified separately (Lambertenghi-Deliliers *et al.*, 1991). MDS were considered as primary in the absence of previous bone marrow disorder or treated neoplasia. MDS were considered as secondary when they were diagnosed after a previous treated neoplasia or an haematological malignancy.

Detailed distribution of the population by age and sex provided by the National Institute for Statistics and Economic Studies (INSEE) was used to calculate incidence rates. For the purpose of regional comparison, rates were standardised by the direct method using the World Standard Population. For the comparison of rates in urban and rural areas within Côte d'Or the so-called indirect standardisation method was used. The standardised incidence ratio (SIR) was calculated as the ratio of observed cases *vs* expected cases. Transformation rates and survival rates were calculated using the life-table method. Corrected survival rates were calculated, these being defined as the ratio of the observed survival rates and the expected survival rates derived from the French population life-tables. Transformation and survival curves were compared by means of the log-rank test. The health status of all patients was updated in December 1993.

### Results

### *Incidence*

A total of 167 MDS were diagnosed among Côte d'Or residents between 1980 and 1990. They represented 9.5% of the 1754 registered cases of HM. The crude incidence rate was 3.2 per 100 000 per year (3.8 in men and 2.5 in women). The corresponding age-standardised rate was 1.7 per 100 000 per year (2.3 in men and 1.2 in women) (sex ratio, 1.9). Age-specific incidence rates are given in Figure 1. There was a male predominance in all age groups. MDS were rare before the age of 60 (12%). After 60 incidence rose rapidly with age,

Correspondence: M Maynadié, Laboratoire d'Hématologie, Hôpital du Bocage, BP 1542, 21034 Dijon Cedex, France
Received 22 December 1995; revised 1 February 1996; accepted 5 February 1996

more steeply in men than women. The mean age was 73 for men and 74 for women (NS). The risk of MDS was higher in urban than in rural areas. For men the SIR was respectively 1.31 and 0.60 ($P<0.01$). The corresponding figures for women were 1.18 and 0.71 (NS).

RAEB was the most frequent subtype of MDS (33%) before RARS and CMML (21% each), RAEBt (13%), RA (8%) and MD with MF (4%). Analysis by sex showed a male predominance in all subtypes except for RA and MD with MF (Table I). RA was diagnosed in relatively younger patients than other MDS cases (three cases were diagnosed before 40 years) (mean age 64 years). On the other hand, CMML was diagnosed later in life (mean age 78 years). Mean age for other MDS cases were 69 years for MD with MF, 71.5 years for RAEBt, 72 years for RAEB and 76 years for RARS. Overall, 146 MDS were classified as primary (87%) and 21 as secondary (12.5%).

There was no significant change in incidence over the studied period. Age-standardised rates for 100 000 inhabitants were 1.5 for the 1980–82 period, 1.4 for the 1983–85 period, 2.0 for the 1986–88 period and 1.7 for the 1989–90 period. Rates by 3 year period and by sex revealed a 1.5-fold increase in incidence in men between the 1980–82 (1.8) and the 1986–88 (2.7) periods but a stable rate in the 1989–90 period (2.6). In women incidence rates did not increase, they were 1.2 for the 1980–82 period, 0.1 for the 1983–85 period, 1.6 for the 1986–88 period and 1.1 for the 1989–90 period.

### *Progression and survival*

Overall 5 year transformation rate of MDS was 31% (±4%) but it was different according to the subtype. For RAEBt the 1 year transformation rate was 44% and the 5 year rate was 100%. In contrast no transformation occurred in MD with MF during the study period. For other MDS subtypes the 5 year transformation rate was 39% (±8%) for RAEB, 30% (±9%) for CMML, 7% (±5%) for RARS and 17% (±11%) for RA. All the transformed MDS were myeloid leukaemias. Among them 54% were M2 FAB subtype (20/37), 16% were M4 (4/37), 13% were M1 (5/37), 8% were M5 (3/37), 5% were M0 (2/37) and 3% were M6 (1/37) but none were M3 FAB subtype. The observed 5 year survival rate was 23%±3% and the corresponding corrected rate was 33%. Corrected 5 year survival rate was 0 for RAEBt, 24% for RAEB, 33% for CMML, 34% for MD with MF, 35% for RARS and 50% for RA. The prognosis of RAEBt was worse than the prognosis of other subtypes ($P<0.01$). Corrected survival curves according to the subtype of MDS are given in Figure 2.

**Figure 1** Age-specific incidence rates of MDS subtype per 100000 inhabitants per year in Côte d'Or. –◆–, men; –x–, women.

**Table I** Distribution of cases of MDS by subtype diagnosed in Côte d'Or between January 1980 and December 1990

| | *Number of cases* | | |
|---|---|---|---|
| *FAB subtype* | *M* | *F* | *Total (%)* |
| RA | 5 | 8 | 13 (8%) |
| RARS | 22 | 13 | 35 (21%) |
| RAEB | 32 | 23 | 55 (33%) |
| RAEB t | 12 | 10 | 21 (13%) |
| CMML | 27 | 8 | 35 (21%) |
| MD with MF | 2 | 5 | 7 (4%) |
| Total | 100 | 67 | 167 |

MDS, myelodysplastic syndromes; RA, refactory anaemia; RARS, refractory anaemia with ring sideroblasts; RAEB, refractory anaemia with excess of blasts; RAEB t, refractory anaemia with excess of blasts in transformation; CMML, chronic myelomonocytic leukaemia; MD with MF, myelodysplastic sydromes with myelofibrosis. M, male; F, female.

### Discussion

Very little data are available on the incidence of MDS. Most of it concerns the United Kingdom (UK) (Cartwright *et al.*, 1990; Williamson *et al.*, 1994; Phillips *et al.*, 1994). Incidence rates have also been published for the Düsseldorf area (Aul *et al.*, 1992). The reported data describe, for the first time, the epidemiological characteristics of MDS in a well-defined French population.

The incidence of MDS in France appears to be close to the incidence in Düsseldorf (4.1 per 100 000) or in England and Wales (3.6 per 100 000) (Aul *et al.*, 1992; Cartwright *et al.*, 1990). In the UK there are important variations in incidence according to areas. Very high incidence rates have been reported in east Dorset (12.6 per 100 000) and Somerset (9.3 per 100 000) (Williamson *et al.*, 1994; Phillips *et al.*, 1994). This discrepancy does not seem to be attributable to incomplete registration. Many reasons could explain these differences. First of all the proportion of elderly people, in whom MDS are more frequent, was higher in east Dorset (22.5% were over 65) and in Somerset (25% were over 60) than in our population (13.5% were over 65). The second explanation mentioned was the well-established health-screening of the elderly in these two regions. As 42% of MDS have been diagnosed in the event of an incidental blood test in our area, more systematic blood tests in the elderly population could explain part of the observed differences. Furthermore, certain peripheral blood findings are difficult to diagnose even for a specialist. In such cases only a bone marrow examination can detect a MDS. Williamson *et al.* (1994) made strenuous efforts to document new cases by adopting a low threshold for performing marrow examination in patients with suggestive peripheral blood findings. It is

**Figure 2** Corrected survival rates of each subtype of MDS. –◆–, refractory anaemia; –*–, refractory anaemia with ring sideroblasts; –x–, refractory anaemia with excess of blasts; –▲–, refractory anaemia with excess of blasts in transformation; –●–, chronic myelomonocyte leukaemia.

very probable that in some areas a bone marrow examination was not provided in all patients, especially when they are asymptomatic or stable or very old. For these reasons we think that real incidence of MDS is higher than that generally reported.

The well-established health screening of the elderly in Dorset could also partly explain the repartition of MDS subtypes diagnosed in their population. They noted a higher proportion of often asymptomatic and quite stable MDS as RA and CMML. Consequently RAEB and RAEBt are less numerous. The greatest discrepancy is the number of RA: we found only 8% of RA compared with 21% and 43% in the other studies (Cartwright *et al.*, 1990; Aul *et al.*, 1992; Williamson *et al.*, 1994). An explanation could be the harshness of our criteria for including RA. In addition to a regenerative anaemia with dyserythropoietic features in bone marrow, we required at least one other biological symptom of MDS such as bone marrow haematopoietic progenitors, abnormal *in vitro* growth pattern or abnormal karyotype at diagnosis or during the follow-up. In fact RA is the most difficult MDS to diagnose even for a specialist because of the paucity of objective characteristics and we think that it is often overevaluated. Other subtypes are as frequent in England and Wales as in France.

The male predominance as found in our study had already been pointed out (Cartwright *et al.*, 1990; Aul *et al.*, 1992). No previous report has examined urban and rural differences. Cartwright reported geographical variations in the UK but did not consider urban/rural variations (Cartwright *et al.*, 1990). The explanation for the higher incidence in urban areas is unknown; it may be the result of some unidentified occupational or environmental exposure or some artefact of the way the data were collected. In our register, the proportion of secondary MDS, due to previous chemotherapy amounted to 12.5% of all MDS.

A discrepancy between England and Germany and France concerns the trends. Important increases in incidence have been reported in England: a 2-fold increase between 1984 and 1988 and in Germany a 3-fold increase in incidence between the 1976–80 and the 1986–90 periods (Cartwright *et al.*, 1990; Aul *et al.*, 1992). In France incidence rates increased but not so rapidly at the beginning of the decade and have remained stable since 1988–1989 (Figure 1). In Düsseldorf, they also noted that the rates are quite stable in the later years studied (Aul *et al.*, 1992). The increase generally observed coincided with the publication in 1982 of the FAB classification, which was responsible for a better knowledge and an easier diagnosis of MDS (Bennett *et al.*, 1982).

No results have been published so far concerning transformation rate in population-based statistics. Our data allow consideration of three groups: RAEBt with a high risk of transformation, which is nearly 50% 1 year after the diagnosis and 100% at 5 years, MD with MF and RA with the lower risk with a 5 year cumulative transformation rate being less than 10%, and RAEB, CMML and RARS with an intermediate 5 year risk ranging between 18% and 32%. Similar results have been reported in hospital-based statistics (Kerkhofs *et al.*, 1987).

Survival analysis confirms the worse prognosis of RAEBt. RARS, MD with MF and CMML are equivalent in terms of survival. CMML survival rate is close to some reports with a median survival time of 50 months and different from other studies in which it is worse (Fenaux *et al.*, 1987; Mufti *et al.*, 1985; Tricot *et al.*, 1984). This discrepancy could be owing to selection bias in a haematological centre, as opposed to population-based registries which include all diagnosed cases. In RARS the 5 year transformation rate was quite low whereas the survival rate was bad. The evolution of each MDS subtype was different and the follow-up and the therapeutic attitude have to be adjusted. Epidemiological studies are necessary not only to establish epidemiological features useful for public health attitudes but also to define prognosis factors and generate suitable therapeutic schemes. This is particularly true in western countries where the elderly population is becoming more and more important.

**Acknowledgements**

We thank Professor Jean Faivre for his helpful criticism and Mrs J Milan for her help in statistical work. The Registre des Hémopathies Malignes de Côte d'Or benefits from a research grant from Comité Départmental of the Ligue Nationale Contre le Cancer.

### References

AUL C, GATTERMANN N AND SCHNEIDER W. (1992). Age-related incidence and other epidemiological aspects of myelodysplastic syndromes. *Br. J. Haematol.*, **82,** 358–367.

BENNETT JM, CATOVSKY D, DANIEL MT, FLANDRIN G, GALTON DAG, GRANILCK HR AND SULTAN C. (1982). Proposals for the classification of the myelodysplastic syndromes. *Br. J. Haematol.*, **51,** 189–199.

CARLI PM, MILAN C, LANGE A, DEVILLIERS E, GUY H AND FAIVRE J. (1986). Haematopoietic malignancies in Côte d'Or (France): a population based study. *Br. J. Cancer*, **53,** 811–815.

CARTWRIGHT RA, ALEXANDER FE, MCKINNEY PA AND RICKETTS TJ. (1990). Myelodysplastic states. In *Leukemia and Lymphoma. An Atlas of Distribution within Areas of England and Wales (1984–1988)*. pp. 32–40. Leukemia Research Fund: London.

FENAUX P, JOUET JP, ZANDECKI M, LAZI JL, SIMON M, POLLET JP AND BAUTERS F. (1987). Chronic and subacute myelomonocytic leukemia in the adult: a report of 60 cases with special reference to prognostic factors. *Br. J. Haematol.*, **65,** 101–106.

INSTITUT NATIONAL DE LA STATISTIQUE ET DES ETUDES ECONOMIQUES (INSEE). (1991). *Resencement Général de la Population de 1990*. INSEE: PARIS.

JANSSEN JWG, BUSCHLE M, LAYTON M, DREXLER HG, LYONS J, VAN DEN BERGHE H, HEIMPEL H, KUBANEK B, KLEIHAUER E, MUFTI GJ AND BARTRAM CR. (1989). Clonal analysis of myelodysplastic syndromes: evidence of multipotent stem cell origin. *Blood*, **73,** 248–254.

KERKHOFS H, HERMANS J, HAAK HL AND LEEKSMA CHW. (1987). Utility of the FAB classification for myelodysplastic syndromes: investigation of prognostic factors in 237 cases. *Br. J. Haematol.*, **65,** 73–81.

LAMBERTENGHI-DELILIERS G, ORAZI A, LUKSCH R, ANNAROLO C AND SOLIGO D. (1991). Myelodysplastic syndrome with increased marrow fibrosis: a distinct clinico-pathological entity. *Br. J. Haematol.*, **78,** 161–166.

MUFTI GJ, STEVENS JR, OSCIER DG, HAMBLIN TJ AND MACHIN D. (1985). Myelodysplastic syndromes: a scoring system with prognostic significance. *Br. J. Haematol.*, **59,** 1425–1433.

PHILLIPS MJ, CULL GM AND EWINGS M. (1994). Establishing the incidence of myelodysplasia syndrome. *Br. J. Haematol.*, **88,** 896–897.

REIZENSTEIN P AND DABROWSKI L. (1991). Increasing prevalence of the myelodysplastic syndromes. An international Delphi study. *Anticancer Res.*, **11,** 1069–1070.

TRICOT G, DE WOLF-PETERS C, HENDRICKX B AND VERWILGHEN RL. (1984). Bone marrow histology in myelodysplastic syndromes. I. Histological findings in myelodysplastic syndromes and comparison with bone marrow smears. *Br. J. Haematol.*, **57,** 423–430.

WILLIAMSON PJ, KRUGER AR, REYNOLDS PJ, HAMBLIN TJ AND OSCIER DG. (1994). Establishing the incidence of myelodysplastic syndrome. *Br. J. Haematol.*, **87,** 743–745.

WORLD HEALTH ORGANIZATION (WHO). (1977). *International Classification of Diseases*. 9th revision. World Health Organization: Geneva.